# Advancing the frontier of left ventricular assist device outflow graft stenting: Stenting techniques and outcomes in a landmark case series

**DOI:** 10.1016/j.jhlto.2025.100235

**Published:** 2025-02-20

**Authors:** David Gittess, Mohammad A. Al-Ani, Andrew Stein, Juan R. Vilaro, Alex M. Parker, Juan M. Aranda, Mustafa M. Ahmed, Eric Jeng, R. David Anderson

**Affiliations:** a Division of Internal Medicine, University of Florida, Gainesville, Florida; b Division of Cardiovascular Medicine, University of Florida, Gainesville, Florida; c Division of Cardiothoracic Surgery, University of Florida, Gainesville, Florida

**Keywords:** heart failure, left ventricular assist device, outflow graft obstruction, interventional cardiology, percutaneous intervention

## Abstract

**Background:**

Left ventricular assist devices (LVADs) have demonstrated a significant mortality and quality-of-life benefit to patients with end-stage heart failure. However, a unique potential complication is outflow graft obstruction (OGO), in which the conduit between the LVAD pump and the aorta is narrowed. Recently, attempts at an endovascular approach with outflow graft stenting have been successful with comparatively low risk relative to surgical replacement of the LVAD. Because of the rarity of this event, optimal stenting technique and periprocedural management are unclear.

**Methods:**

In this series of 10 patients, we detail the percutaneous endovascular approach to OGO treatment. All cases were confirmed with contrasted computed tomography.

**Results:**

One patient experienced OGO recurrence and one patient had 2 recurrences, making the total interventions analyzed 13. The obstructions were either intrinsic, with accumulation of biodebris between the graft and the outer bend relief in 9 patients, or extrinsic from outflow graft kinking in 4 patients. All patients underwent successful outflow graft stenting confirmed by rapid restoration of pre-obstruction flow (12/13) or elimination of a pressure gradient across a site of suspected obstruction (1/13). Recurrence was observed in 3 instances. We further describe the stenting technique employed and how the unique nature of OGOs impacts the endovascular approach, and post-procedural outcomes.

The cornerstone of heart failure therapy is medical management; however, advanced therapies exist for patients with end-stage disease who fail to respond to medical therapy. Left ventricular assist devices (LVAD) are associated with a 48% reduction in all-cause mortality,^1^, ^2^ with significant improvement in functional capacity and quality of life.^2^ In 2022, 1-year survival for an LVAD patient was 83%, with 5-year survival at 52%.^3^ However, a unique complication of LVAD support is outflow graft obstruction (OGO). It commonly occurs due to accumulation of biodebris between the outflow graft and outer bend relief sheath, and it can be associated with the porousness of the graft material and the length of the graft.^4^ External factors such as compression or kinking of the outflow graft can play a role as well.^4^, ^5^, ^6^ LVAD OGOs are rare events; in a large international multicenter retrospective analysis, Wert et al. found an incidence of biodebris-related-OGO in 3.0% of HeartMate 3 devices among 2,108 patients.^7^ This incidence increased with longer duration of LVAD support, peaking at a 5-year incidence of 9.1%, and it also varied considerably among the study centers, from 0 instance of OGO at one institution to 12.1% of LVADs affected in another.^7^ Other prior case series have documented an incidence ranging from around 1–6% of all LVAD patients.^8^, ^9^, ^10^

Hemodynamically, OGOs result in high LVAD afterload which can lead to low pump flow, pump thrombosis, arterial emboli, decompensated heart failure, worsening mitral insufficiency, arrhythmia, or combination of the above. Given the above, symptomatic OGOs represent a significant complication and may need prompt treatment.^11^ As the LVAD cohort grows, with increasing destination therapy use as well as improved survival and longer periods of durable support, OGO incidence will likely continue to increase in the near future.^10^

Best practices for OGOs management remain unknown as this is an emerging condition with variable diagnostic techniques and therapeutic approaches between individual centers. Recently, case reports and series have begun to show the value of an endovascular approach to treatment of OGO.^12^, ^13^, ^14^, ^15^, ^16^, ^17^, ^18^ Such approaches differ between institutions, and there’s a literature gap in interventional planning.

In this case series, we further investigate a percutaneous endovascular approach and present one of the largest available OGO stenting cohorts. Our goal is to describe our center’s standardized approach to OGO endovascular stenting technique and our intervention outcomes.

## Methods

This case series represents a single-center retrospective chart review. Patients were included if they had received an LVAD between June 1, 2010, and January 1, 2023, had an OGO, and received stenting of the OGO for therapeutic management. Patients who met these inclusion criteria were evaluated for the following: clinical course prior to OGO diagnosis, method of diagnosis, and outcome including return of LVAD flow (liter/min) and re-occlusion of the graft. The data were analyzed with descriptive statistics. The study was approved by the institutional review board (IRB202202763), and informed consent was waived due to the anonymous review and minimal risk to participants.

## Results

Ten out of 529 LVAD patients met all inclusion criteria, accounting for an incidence of OGO at 1.9%. Patients were on average 50.5 years old (median 45 years old) at the time of their first OGO. Nine patients (90%) were male; by race, 6 were black/African American, 3 were white, and 1 was Hispanic. Eight of 10 patients had non-ischemic cardiomyopathy (NICM). By device, 4 patients had a Heartmate 2, 4 patients had a Heartmate 3, and 2 patients had a Heartware device. All VAD implantations were performed via full median sternotomy and only 2 patients had a prior sternotomy: one for a VAD exchange after acute hemodynamic changes from an outflow graft thrombus and the other for coronary artery bypass surgery (Table 1).

**Table 1:** Demographics

Record	Age	Sex	Race	Type of cardiomyopathy	Type of VAD	Prior sternotomy	Pump exchange prior to stenting
1	55	M	Black	Ischemic	HM3^a^	No	No
2a^b^	45	M	Black	Non-ischemic	HM3	No	No
2b^b^	45					No	No
3	48	M	Hispanic	Non-ischemic	Heartware	No	No
4	59	M	Black	Non-ischemic	HM2^c^	Yes – done to place first LVAD	Yes – exchanged because of outflow graft thrombus
5	47	M	Black	Non-ischemic	HM3	No	No
6a^b^	40	M	Black	Non-ischemic	Heartware	No	No
6b^b^	41					No	No
6c^b^	44					No	No
7	34	M	White	Non-ischemic	HM2	No	No
8	44	F	Black	Non-ischemic	HM2	No	No
9	83	M	White	Non-ischemic	HM2	No	No
10	50	M	White	Ischemic	HM3	Yes – CABG^d^	No

LVAD, left ventricular assist device; OGO, outflow graft obstruction.

a HM3: Heartmate 3.

b Patients 2 and 6 had multiple OGOs treated with stenting.

c HM2: Heartmate 2.

d CABG: coronary artery bypass graft.

The majority of patients presented with symptomatic heart failure secondary to low LVAD flow (85% or 11/13 total obstruction events) (Table 2). The diagnosis was universally suspected or confirmed by computed tomography (CT) scanning, of which 77% were done under a cardiac angiography protocol and the remaining 23% on non-gated CT chest with contrast. Obstructions were most commonly due to intrinsic compression from biodebris between the graft and the outer Gore-Tex layer (9/13, 69%). The remaining obstructions (4/13, 31%) were extrinsic, due to kinking of the entire graft structure. The median amount of time between LVAD implantation and first obstruction was 1,113 days (inter-quartile range of 1,068 days), and the median amount of time between LVAD implantation and first stenting was 1,120 days (inter-quartile range of 1,040 days).

**Table 2:** Clinical Characteristics

Record	Presentation	How diagnosis was made	Type of obstruction	Days from VAD implant to obstruction diagnosis	Days from VAD implant to stenting
1	dyspnea with elevated filling pressures on RHC, decreased LVAD flow	CT angiography aorta	intrinsic – concentric mural thrombus	853	874
2a	low flow alarms	Initial RHC with speed optimization suggestive of OGO, confirmed by later CT angiography aorta	intrinsic – thrombus	1,360	1,379
2b	low flow alarms	CT chest	extrinsic – twisting and compression of outflow graft	37 days since prior OGO stenting	37 days since prior OGO stenting
3	low RTFs	Initial RHC ramp study suggestive of abnormal hemodynamics, with OGO confirmed by CT angiography	intrinsic – moderate stenosis in mid-cannula and severe stenosis in cranial aspect connecting to aorta	779	819
4	SOB, low flow alarms	CT angiography	intrinsic – eccentric thrombus throughout the entire outflow cannula with severe luminal narrowing in the mid cannula	3,029	3,033
5	low flow alarms, elevated LDH, and acute renal dysfunction	CT angiography cardiac structures	intrinsic – large amount of thrombus adjacent to the aortic cannulation site causing significant stenosis	1,865	1,873
6a	low flow alarms	CT chest	extrinsic – unspecified cause	797	833
6b	low flow alarms, fatigue	CT angiography	extrinsic – kinking compression around fractured stent	119 days since prior OGO stenting	119 days since prior OGO stenting
6c	low flow alarms, volume overload	CT angiography cardiac structures	Intrinsic severe stenosis of the proximal graft likely in the site of a previously stented area	1,117 since prior OGO stenting	1,117 since prior OGO stenting
7	low flows on interrogation	CT angiography aorta	extrinsic – unspecified cause	1,169	1,178
8	low flow alarms	Initial RHC with ramp study suggestive of device malfunction, confirmed by CT angiography cardiac structures	intrinsic – interval increased thrombus with near complete occlusion of the mid portions of the LVAD	1,057	1,062
9	incidental finding	Incidental finding on CT intravenous pyelogram and confirmed on CT chest	intrinsic – mural thrombus involving the inflow and outflow cannula of the LVAD	3,134	3,579
10	abnormal hemodynamics on right heart catheterization	Initial RHC with ramp study suggestive of OGO, confirmed by CT angiography cardiac structures	Intrinsic obstruction of the outflow graft	305	324

CT, computed tomography; LDH, lactate dehydrogenase; LVAD, left ventricular assist device; OGO, outflow graft obstruction; RHC, right heart catheterization; RTF, return to flow; SOB, shortness of breath.

Regarding medical management (Table 3), every patient except one was on an anti-platelet agent, the most common being aspirin monotherapy (8/10 pre-stenting and 7/10 post-stenting). Post-stenting, 3 patients were started on clopidogrel, and warfarin dose adjustments were common. One person was initiated on apixaban for anticoagulation. The issue of antiplatelet and anticoagulation agents after stenting is an important one. In 2 instances, the antiplatelet therapy was de-escalated; however, in both of these cases the anticoagulation therapy was increased.

**Table 3:** Medical Management Before and After OGO Stenting

	OGO pre-stenting medical management		OGO post-stenting medical management	
Record	Anti-thrombotic	Anti-coagulation	Anti-thrombotic	Anti-coagulation
1	aspirin	warfarin	same	same
2a	aspirin, dipyridamole	warfarin	clopidogrel for 3 months followed by aspirin	warfarin (increased from admission)
2b	clopidogrel	warfarin	same	same
3	aspirin	warfarin	clopidogrel	warfarin (increased from admission)
4	aspirin	warfarin	same	warfarin (increased from admission)
5	aspirin	warfarin	same	warfarin (increased from admission) with enoxaparin bridging
6a	aspirin	warfarin	aspirin, clopidogrel	warfarin (decreased from admission)
6b	aspirin	warfarin	same	warfarin (increased from admission)
6c	aspirin	warfarin	same	warfarin (decreased from admission)
7	aspirin	warfarin	same	same
8	aspirin	warfarin	same	warfarin (increased from admission)
9	n/a	warfarin	n/a	same
10	aspirin, ticagrelor	n/a	aspirin	apixaban

OGO, outflow graft obstruction.

### Outflow graft obstruction stenting technique and outcomes

Eleven of 13 catheterizations obtained access through a femoral artery (85%). One obtained access through the right radial artery, and the remaining used bilateral radial arteries. The average fluoroscopy time among the 9 cases with recorded duration was 34.04 minutes, though it ranged from 25.4 minutes to 61.4 minutes. Intravascular ultrasound (IVUS) was used in 85% of approaches; these studies were done on a Phillips Volcano system. Angiography was employed pre-stenting in 46% of cases and post-stenting in 77% of cases. All instances involving angiography used Omnipaque contrast with an average dose of 143.4 ml (median 137.5 ml). The most common immediate complications were small access site hematomas (3/13, 23%), though 1 patient experienced a cardiac arrest in the catheterization lab and 1 patient had a post-procedural stroke that was treated with thrombectomy. Both made full recoveries. Other technical details are recorded in Table 4.

**Table 4:** Technical Details and Equipment

Record	Type of intervention	Access site	Access equipment	Stenting equipment used	Fluoroscopy time	IVUS	Angiography	Contrast media and dose	Immediate complications
1	Left ventriculograms showed OGO, confirmed with IVUS; initial failure with stenting; balloon angioplasty allowed for re-attempt at stenting, which was successful	R femoral artery	Proglide, Cook sheath, JR4 catheter, Storq guidewire, Amplatz super stiff guidewire	Vici 16 × 90 mm >> Atlas balloon 16 × 40 mm x2 >> iCast 10 × 38 mm bare metal stent >> Atlas balloon 16 × 40 mm	26 minutes, 24 seconds	Yes	Yes – after stenting to confirm placement	Omnipaque 145 ml	access site hematoma
2a	IVUS showing compression followed by stenting which was unsuccessful, so more stents deployed	R femoral artery	Cook sheath, JR4 catheter, Storq wire, Amplatz super stiff wire	Viabahn VBX 11 × 79 mm x4 and 11 × 39 mm x1, followed by Viabahn VBX 9 × 79 mm x3	61 minutes, 24 seconds	Yes	Yes – after stenting to confirm placement	Omnipaque 25 ml	access site hematoma requiring evacuation
2b	IVUS showing compression followed by stenting	L femoral artery	Cook sheath, JR4 catheter, Storq wire, Amplatz super stiff wire	Viabahn VBX 11 × 19 mm x2 and 11 × 59 mm x1	27 minutes, 0 second	Yes	Yes – before stent deployment	Omnipaque 118 ml	ischemic stroke treated with thrombectomy; no residual deficits
3	Ascending aortography showed obstruction; stenting followed without improvement; angiography and IVUS showed new obstruction and 2 additional stents were placed with return of flow	R femoral artery	MicroWire, Storq wire, Shuttle sheath, JR4 catheter, Amplatz super stiff wire	Viabahn VBX 10 × 79 mm stent x1, followed by the same stent x2	unspecified	Yes	Yes – before and after stent deployment	unspecified	n/a
4	Graft angiography, stenting without improvement in flow; IVUS identified another stenosis that was treated with stenting	R femoral artery	Proglide, MP1 catheter, Storq guidewire, Cook catheter, Amplatz super stiff guidewire	VBX 9 × 79 mm x2, VBX 9 × 39 mm x1 followed by VBX 8 × 79 mm x2	40 minutes, 36 seconds	Yes	Yes – after stenting to confirm placement	Omnipaque 130 ml	n/a
5	IVUS showed narrowing in proximal segment of graft that was stented; confirmatory angiography showed new obstruction in distal segment that was then also stented	R femoral artery	Proglide, Cook sheath, MP1 catheter, Storq guidewire, Amplatz super stiff guidewire	Viabahn VBX 8 × 79 mm x1, followed by another of the same stent	25 minutes, 24 seconds	Yes	Yes – after stenting to confirm placement	Omnipaque 182 ml	access site hematoma
6a	IVUS showed narrowing that was stented; repeat IVUS showed compression near distal anastomosis so another stent place	R radial artery	Storq wire, Cook sheath, Amplatz super stiff wire	Viabahn VBX 10 × 79 mm x3, noncovered Fluency stent 10 × 29 mm x1	unspecified	Yes	Yes – before and after stent deployment	unspecified	n/a
6b	Angiography and pressure measurements used because IVUS could not reach the distal graft; initial stenting resulted in only moderate improvement because of kinking, so second round of stenting followed	R femoral artery	LCB catheter, Storq wire, Amplatz super stiff wire	iCAST 9 × 58 mm x1, followed by iCAST 9 × 58 mm x1 and 9 × 38 mm x1	unspecified	Yes	No	n/a	n/a
6c	Pressure measurements because IVUS could not reach distal portion of the graph; followed by stenting	R femoral artery	Proglide, MP catheter, Storq wire, Cook catheter, Amplatz super stiff, JR4 catheter	Viabahn VBX 9 × 39 mm x1 and 9 × 79 mm x1	31 minutes, 12 seconds	Yes	Yes – after stenting to confirm placement	Omnipaque 150 ml	n/a
7	Angiography was unsuccessful; stenting performed but did not fully relieve compression, so more stenting done	Bilateral radial arteries	R wrist - Storq wire; L wrist - Cook sheath, JR4 catheter, Amplatz super stiff wire	Zilver PTX 14.0 mm x3, followed by iCAST 10 × 37 mm and 9 × 57 mm overlapping	unspecified	Yes	Yes – before and after stent deployment	unspecified	n/a
8	IVUS followed by stenting; however, this did not relieve distal obstruction and led to "watermelon seeding" of lesions throughout graft that was further complicated by distortion of stent; additional stenting performed	L femoral artery	Proglide, Cook sheath, JR4 catheter, Storq guidewire, Amplatz super stiff guidewire	Zilver 14 × 60 mm x2, followed by iCast 10 × 38 mm covered bare metal stents x2	36 minutes, 18 seconds	Yes	Yes – before and after stent deployment	Omnipaque 291 ml	n/a
9	Elective procedure for non-obstructive outflow graft; angiography and pressure measurements followed by stenting x2; subsequently, patient became hypotensive with concern for obstructive shock from the catheter sheath; patient went into VT arrest but was resuscitated and a third stent placed	R femoral artery	MP catheter, Storq wire, Cook sheath	VBX 8 × 79 mm and 8 × 59 mm x1, followed by an additional 8 × 59 mm stent	29:06:00	No	Yes – before stent deployment	Omnipaque 106 ml	intra-procedural VT arrest necessitating post-cardiac arrest care
10	Uncomplicated stenting; flows did not improve but pressure gradient across MP catheter went to zero, suggestive of successful resolution of OGO	R femoral artery	MP catheter, Storq wire, Cook sheath, Amplatz super stiff wire	VBX 11 × 39 mm x2, 9 × 79 mm x3, 8 × 79 mm x1	29:00:00	No	No	n/a	n/a

IVUS, intravascular ultrasound; OGO, outflow graft obstruction; VT, ventricular tachycardia.

**Figure 1:**
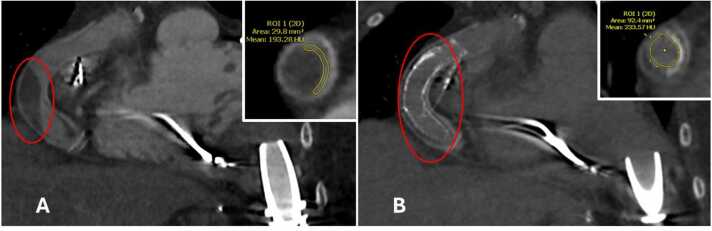
CT chest pre- and post-stenting. (A) CT chest pre-stenting. Area of minimal flow of 29.8 mm^2^. LVAD flow of 2.5 liter/min. (B) CT chest post-stenting. Area of minimal flow of 92.4 mm^2^. LVAD flow of 5 liter/min. CT, computed tomography; LVAD, left ventricular assist device.

Outcomes are reported in Table 5. In only 23% (3/13) of catheterizations, the first stents used were fully successful. The remaining 77% (10/13) were complicated by the appearance of other stenoses within the outflow graft immediately after the first stents were placed. After this first cardiac catheterization event, 80% were fully treated; only 2 patients (20%) developed re-stenosis that required a second attempt at catheterization. In 92% of stenting events (12/13), normal or improved LVAD hemodynamics were noted after initial stenting. In the final patient, LVAD flow improved by 0.4 liter/min but remained abnormal; however, the pressure differential measured across the stent at the area of obstruction was 0, indicating patency. His LVAD speed was then safely reduced by 700 rpm without compromising LVAD flow. One patient’s stenting was also notable for intra-procedural cardiac arrest likely due to prolonged outflow graft occlusion during stenting. The patient was resuscitated, and stenting was completed. This patient was also notable as he was the only one whose OGO was discovered incidentally; thus, he was also the only patient for whom stenting was initially considered elective. Two patients later went on to transplant, but the pathology report did not comment on findings relevant to OGO obstruction. Every patient had full resolution of their presenting symptoms. Figure 1 demonstrates an example of successful therapy based on CT findings.

**Table 5:** Outcomes

Record	Outcome	Pre-procedure flow (liter/min)	Post-procedure flow (liter/min)	Symptom change	Restenosis (Y/N)	Pathology (if patient went on to transplant)
1	increase in flow	4 (as of 3 weeks prior)	4.3	resolution of dyspnea and headaches	No	none
2a	increase in flow	3.3	4.6	planned admission; was asymptomatic at presentation	Yes	none
2b	increase in flow; follow up imaging showed no change from before second PCI	3.3	5.1	planned admission; was asymptomatic at presentation	No	unremarkable
3	return of flow by angiography, no stenosis by IVUS	2.5	6	planned admission; was asymptomatic at presentation	No	none
4	return of flow by angiography	4.4	5	resolution of dyspnea	No	none
5	return of normal VAD hemodynamics	3.7	5	resolution of fluid overload	No	none
6a	return of normal VAD hemodynamics	0.6	5.5	resolution of dizziness, low flow alarms	Yes	none
6b	return of normal VAD hemodynamics	3.7	5.8	resolution of fatigue	Yes	none
6c	return of normal VAD hemodynamics	2.9	7	resolution of fluid overload	No	unremarkable
7	return of normal VAD hemodynamics	2.5	5	resolution of cardiogenic shock and respiratory failure	No	none
8	return of normal VAD hemodynamics	2.5	5	resolution of fatigue and low flow alarms	No	none
9	return of normal VAD hemodynamics	4.2	5.5	resolution of dyspnea, fluid overload, fatigue	No	none
10	no change in VAD flow but pressure gradient across obstruction fell to zero and speed was safely reduced without compromising flow	5.5	5.9	successful resuscitation after cardiac arrest	No	none

IVUS, intravascular ultrasound; PCI, percutaneous coronary intervention; VAD, ventricular assist device.

The procedures were overall well tolerated; however, we do note that the recurrence rate for OGO after initial stenting in our cohort was not insignificant (3/13, 23%). This is a relatively small cohort, hence our ability to meaningfully estimate the recurrence rate of OGO obstruction after initial stenting is limited. The most common complications we saw were complications associated with cardiac catheterizations more generally including access site hematomas and major adverse cardiac events, of which we saw one, likely due to prolonged outflow graft occlusion time.

## Discussion

### Outflow graft obstruction stenting carries unique technical challenges

LVAD outflow graft obstructions remain a poorly understood clinical entity with an unclear therapeutic approach. In this case series, we demonstrate 13 successful attempts at percutaneous endovascular stenting, making it one of the largest available in the literature. Our patients presented at 2 extremes: asymptomatic with LVAD alarms or in an acute heart failure decompensation episode. All suspected OGOs were first diagnosed by CT scan or by LVAD pump findings with significant decreases in flow. Regarding etiologies, intrinsic obstructions were almost always described as a focal or concentric debris within the coaxially placed original and protective outflow grafts, suggesting the etiology of proteinaceous, acellular biodebris rather than a hematologic thrombus.

**Figure 2:**
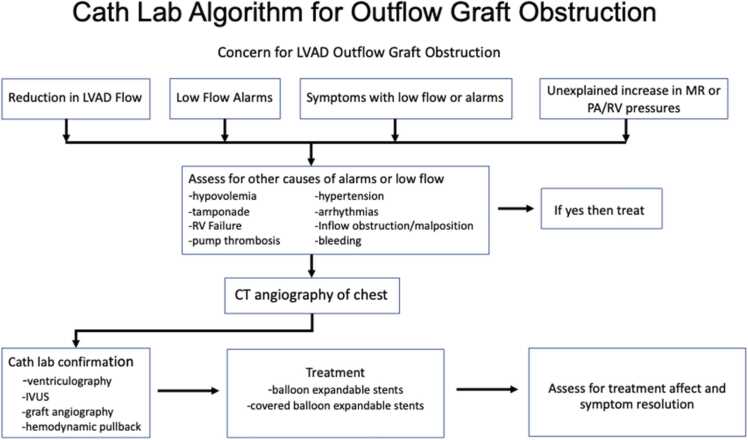
Diagnostic and therapeutic algorithm for management of OGO. OGO, outflow graft obstruction.

Treatment of OGOs evolved over the course of this study. Initially many different attempts at imaging with IVUS, angiography, and LV angiography following into the outflow graft phase were employed. These were all suboptimal in confirming or refuting an actual outflow graft obstruction. Standard IVUS catheters were not long enough to reach the proximal portion of the outflow graft near the LVAD. Angiography was typically poor quality and equivocal in confirming obstruction. We found that the technique that provided the best evidence for an obstruction, even when the CT scan was equivocal, was hemodynamic pullback with a multi-purpose catheter. This universally provided evidence for a pressure drop between the actual LVAD and the aortic anastomosis and was the only technique used in the last several patients. From a surgical perspective, our practice has eliminated the implantation of the surrounding Gore-Tex sheath around the outflow graft, and since we instituted these implant-related techniques we have yet to see an OGO in our cohort of LVAD implantations. An algorithm of the workup and treatment of this condition is presented in Figure 2.

The etiology of the biodebris that occurs between the inner outflow graft and the protective outer graft was not determined pathologically but did easily migrate when a single or 2 stent strategy was tried for treatment. In nearly all cases, this required stenting of the entire graft from the attachment to the LVAD all the way to the aortic anastomosis. In the early experience, different self-expanding stent prostheses were used, but they almost always lacked the radial strength required for an optimal result, usually requiring the addition of ballon expandable stents. Treatment of the last several patients did not involve the use of self-expanding stents and was accomplished exclusively with balloon expandable prostheses (Viabahn VBX – Gore Medical).

Compared to other large studies evaluating percutaneous intervention for outflow graft obstruction, our study shared similar features across patient demographics, medical management, and the intervention approach resulting in successful outcomes, including minimal restenosis. Obstructive etiology was one area of discrepancy, with most of our cases presenting with focal or concentric thrombi in the outflow canula. Agrawal et al. and Nathan et al. both observed a much higher percent of cases (78% each vs 31% in our study) from extrinsic compression or kinking.^10^, ^19^ Regarding technique, Joury et al. developed a slightly different approach, utilizing balloon expanding stents at the most severe stenosis and self-expanding stents to fortify the remainder of the outflow graft.^8^ Meanwhile, Agrawal et al. found themselves stenting the entirety of the graft, similarly to our cases.^10^ Our patients’ mean time from LVAD implantation to presentation was also approximately 3 months delayed compared to the next closest time noted by Joury et al.,^8^ and the longest time to presentation amongst the larger reviews.

A systematic review of HM3 LVAD OGO stenting was conducted by Patil et al., capturing a total of 28 patients.^20^ Similarly to our population, they report that endovascular approaches were overwhelmingly successful (89.3% vs 100% in this case series) with a rapid return of normal LVAD flow rates (85.7% vs 92.3% in this case series).^20^ They noted a rate of recurrent stenosis of 8.0%; in our study, we had 3 episodes of recurrent stenosis accounting for 23.1% of stenting events.^20^ Of note, they also reported external compression and graft twisting as being either the sole or a contributing factor in over half of their patients, with an intraluminal thrombus accounting for 10.7% of events.^20^ Among the 4 HM3 patients in our study, most were due to intrinsic obstructions described as thrombi but more likely proteinaceous debris; only one was diagnosed as secondary to a graft twist.

Curiously, the majority of our patients with outflow graft obstructions were African American, which was the case with 2 other large institutional reviews where race was reported.^8^, ^19^ There is not an obvious explanation for this; however, there may be value in investigating to what degree these reviews demonstrate a trend.

Notably, Abbott Cardiovascular (Pleasanton, California, USA) released a medical device correction in February 2024 to assist with the diagnosis and management of OGOs in HM2 and HM3 models.^21^ They report an incidence of biodebris-related OGO of 0.24% at 2 years and 2.06% at 5 years.^21^ Their recommended workup is a summary of Mehra et al. and focuses on ruling out organic causes for low-flow alarms followed by imaging.^22^ Last – and notably – they include endovascular approaches in their list of therapeutic options.^21^

It is as-of-yet unclear whether certain devices are more prone to OGOs. One study found that Heartmate 2 devices were more susceptible; however it has been the most used LVAD for destination therapy, which could account for increased incidence.^23^ Furthermore, we feel that OGOs occurring with Heartmate 2 devices may be related to the implantation techniques accepted in that era of durable circulatory support. Another study observed a more rigorous graft twisting motion among Heartmate 3 devices compared to others.^24^

An endovascular approach has several strengths that make it a logical option for treatment of OGOs, as it is less invasive, theoretically better than thrombolysis, and associated with low mortality.^23^, ^25^ However, there are noteworthy complications beyond the usual risks associated with endovascular intervention. In one single-center retrospective study, stenting of an obstruction led to outflow graft torsion with critically reduced flows that necessitated urgent surgery.^26^ Periprocedural stroke has been documented in several studies,^8^, ^10^, ^11^, ^26^ and there also exists a theoretical risk of advancing the wire too far into the LVAD pump.

## Conclusions

This case series, like the others, is limited by its small sample size. This makes it difficult to extrapolate and fill in the critical gaps missing in current LVAD management. Moreover, technical expertise and familiarity with the problem grew with more exposure. This could result in stenting events from 2018 having a different outcome or approach than those from 2023.

Further study of this event rate with pooled multicenter experiences that also take into account evolving optimal procedural technique merits further attention. Even in the face of a potentially significant OGO recurrence rate, our experience still suggests OGO stenting likely has a more favorable risk benefit ratios than that associated with open surgical intervention, especially in this population that has inherently high operative risk.

That said, this study adds to the present body of research based upon its size and the critical lessons learned from seeing the problem several times over. Hopefully, this can provide additional clarity to the medical decision-making process when patients present with OGOs.

## Contribution statement

All authors were involved in the conception and design of the project. All authors contributed meaningfully to the manuscript.

## Disclosure statement

Two of the paper’s authors are on the Editorial Board of JHLT-Open: Mustafa M. Ahmed, MD, and Mohammad A. Al-Ani, MD. The authors do not have other disclosures. There are no relationships with industry. There are no conflicts of interest. The authors did not use artificial intelligence to write this article.
